# CAG Student Prize Paper – A3 *ESCHERICHIA COLI* PATHOBIONTS ISOLATED FROM ULCERATIVE COLITIS PATIENTS HARBOUR NUMEROUS PROTEINS THAT DEGRADE HUMAN COLONIC MUCUS

**DOI:** 10.1093/jcag/gwaf042.003

**Published:** 2026-02-13

**Authors:** A Gilliland, A Melville-Bowser, D Tertigas, I Ng, Y Chen, M Surette, B Bressler, B Vallance

**Affiliations:** The University of British Columbia, Vancouver, BC, Canada; The University of British Columbia, Vancouver, BC, Canada; McMaster University, Hamilton, ON, Canada; The University of British Columbia, Vancouver, BC, Canada; The University of British Columbia, Vancouver, BC, Canada; McMaster University, Hamilton, ON, Canada; The University of British Columbia, Vancouver, BC, Canada; The University of British Columbia, Vancouver, BC, Canada

## Abstract

**Background:**

The etiology of ulcerative colitis (UC) remains elusive, with current evidence suggesting weakened mucosal barriers allow noxious luminal stimuli to escape the colonic lumen and trigger inflammation. *Escherichia coli* pathobionts, particularly of phylogroup B2, have been associated with active UC, however their role in disease is unclear. We previously showed that UC-associated *E. coli* p19A can penetrate the mucus barrier of UC air-liquid interface (ALI) monolayers. We hypothesize that *E. coli* pathobionts carried by UC patients degrade their colonic mucosal barriers, thereby contributing to the onset, severity, and chronicity of UC.

**Aims:**

Using healthy and UC patient biopsy-derived colonic organoids and an ALI monolayer model, we investigated the ability of p19A as well as other *E. coli* isolates from UC patients to degrade human colonic mucus.

**Methods:**

ALI monolayers from non-IBD and UC patients were grown, with apical mucus collected, then incubated with secreted proteins from *E. coli* p19A. Degraded mucus was detected by protein gel and MUC2 western blot. To expand our understanding of UC-isolated *E. coli* mucus degradation, we analyzed the phylogeny and the mucinases present in the genomes of 92 *E. coli* strains isolated from UC patients as well as assessed a subset of isolates ability to degrade human mucus.

**Results:**

We show that p19A degrades human ALI monolayer-derived mucus, with UC ALI-mucus more readily degraded. We identified the known mucinases Pic and Vat in p19A and found a Δ*picΔvat* mutant strain retained the ability to degrade mucus, suggesting another secreted protein(s) has mucinase activity. Phylogenetic analysis of 92 clinical *E. coli* isolates revealed 49% are clustered with IBD pathobionts like p19A. *sslE*, *vat*, and *pic* were the most common mucinase genes, present in 54%, 45%, and 30% of the *E. coli* isolates, respectively. In addition, nine *E. coli* isolates derived from four UC patients each harboured three known mucinase genes (*pic*, *vat*, and *sslE*). Mucus degradation assays using secreted proteins from a selected subset of ten phylogenetically distinct isolates (nine from phylogroup B2) revealed that five isolates could degrade both non-IBD and UC-ALI derived mucus, while an additional two isolates could degrade only UC-ALI derived mucus.

**Conclusions:**

UC patients harbor *E. coli* strains that contain numerous mucinase genes, many of which can degrade human ALI monolayer-derived mucus, with UC-ALI mucus more easily degraded. Therefore, specific *E. coli* strains may act as pathobionts, weakening the mucosal barrier in UC patients, thereby contributing to the onset, severity, and/or chronicity of UC.

**Funding Agencies:**

CCC, CIHRWeston Family Foundation (Canadian National Organoid Network)

